# A Rapid and Easy-to-Perform Method of Nucleic-Acid Based Dengue Virus Diagnosis Using Fluorescence-Based Molecular Beacons

**DOI:** 10.3390/bios11120479

**Published:** 2021-11-26

**Authors:** Soumi Sukla, Prasenjit Mondal, Subhajit Biswas, Surajit Ghosh

**Affiliations:** 1 CSIR-Indian Institute of Chemical Biology, 4, Raja S.C. Mullick Road, Kolkata 700032, West Bengal, India; soumi.sukla@niperkolkata.edu.in (S.S.); pmondal@mgh.harvard.edu (P.M.); 2 National Institute of Pharmaceuticals Education and Research, 168, Maniktala Main Road, Kolkata 700054, West Bengal, India; 3 Department of Neurology, Massachusetts General Hospital, Harvard Medical School, Charlestown, MA 02129, USA; 4 Academy of Scientific and Innovative Research (AcSIR), Ghaziabad 201002, Uttar Pradesh, India; 5 Department of Bioscience & Bioengineering, Indian Institute of Technology Jodhpur, NH 62, Surpura Bypass Road, Karwar 342037, Rajasthan, India

**Keywords:** dengue diagnosis, fluorescence, molecular beacon, fluorophore-quencher, ELISA, RT-PCR

## Abstract

Detecting dengue virus (DENV) infection in patients as early as possible makes the disease management convenient. Conventionally, DENV infection is diagnosed by ELISA-based methods, but sensitivity and specificity are major concerns. Reverse-transcription-PCR (RT-PCR)-based detection confirms the presence of DENV RNA; however, it is expensive, time-consuming, and skilled personnel are required. A fluorescence-based detection system that detects DENV RNA in patient’s serum directly, without any nucleic acid amplification step, has been developed. The method uses target-specific complementary sequence in the molecular beacon, which would specifically bind to the DENV RNA. The molecular beacons are approximately 40 bases long hairpin structures, with a fluorophore-quencher system attached at the terminal ends of the stem. These probes are biotinylated in the stem region, so that they can be immobilized on the streptavidin-tagged magnetic beads. These magnetic beads, coupled with biotinylated molecular beacons, are used for the detection of the target RNA in the serum by incubating the mixture. After incubation, beads are separated and re-suspended in a buffer. The measurement of fluorescence is taken in fluorometer after 15 min incubation at 50 °C. The whole work is carried out in a single tube. This rapid method can precisely detect dengue RNA within two hours, confirming ongoing DENV replication in the patient.

## 1. Introduction

**Scheme 1:**
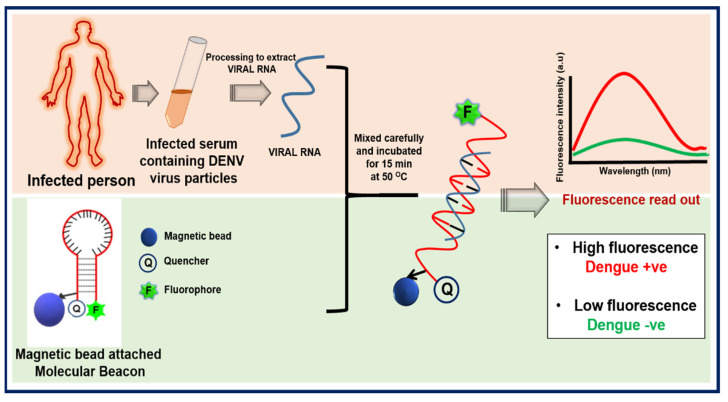
Schematic representation of the functional workflow of the fluorescence quencher system.

Dengue virus (DENV) is the most wide-spread arthropod-borne flavivirus, with an estimated 400 million infections and 100 million symptomatic cases annually, from 128 endemic countries of the world [1]. Another estimate indicates that, on average, 390 million dengue infections occur every year, of which 96 million manifest clinically with any degree of severity of the disease [2]. This highlights the staggering epidemiological and economic burden that is being faced by endemic countries, which includes India and Brazil. DENV generally causes self-limiting dengue fever but can often lead to life-threatening conditions, such as dengue hemorrhagic fever and dengue shock syndrome. The early diagnosis of DENV infection and, thereby, the timely management of the disease, can prevent morbidity and mortality. The ongoing pandemic, due to SARS-CoV-2, has complicated the diagnosis of DENV even more, as there are reports of false-positivity and cross-reactivity between these two viruses, which belong to two different families of viruses [3,4,5,6,7,8,9]. Therefore, in the dengue endemic countries, rapid molecular diagnosis, based on nucleic acid tests, is urgently needed. The methods currently used for the detection of DENV are either antigen/antibody-based ELISAs or nucleic acid-based reverse-transcription-PCR (RT-PCR). The methods of dengue virus diagnosis, using the conventional and biosensor-based methods, have been reviewed in detail [10,11]. To confirm the DENV infection and typing of dengue viruses from clinical samples, RT-PCR is used [12]. A single-reaction, multiplex, real-time RT-PCR for the detection, quantitation, and serotyping of dengue viruses has also been reported [13]. Although ELISA-based methods give fast results, false-negative results are still a concern. The RT-PCR-based method, on the other hand, is time-consuming, expensive, and needs expertise to perform on a routine basis in diagnostic laboratories. Therefore, there is an urgent need of a detection method that can diagnose DENV RNA very fast and with precision (from patient’s samples). Here, in this work, we have described a detection method, which is fluorescence-based and detects DENV RNA in patient’s serum directly, without any nucleic acid amplification step (Scheme 1). This is a rapid method and can precisely detect dengue RNA within two hours, confirming ongoing DENV replication (i.e., active infection) in the patient. This method can also be further improvised to detect different DENV serotypes and other viruses.

## 2. Materials and Methods

### 2.1. Cell Culture

C6/36 cells (NCCS, Pune, India) were used for propagation of the dengue virus. C6/36 cells were cultured in modified Eagle’s medium (Sigma, Bangalore, India), supplemented with 10% FBS (Gibco, TX, USA), Pen-Strep and L-Glutamine (Sigma, Rehovot, Israel) and Fungizone (Gibco). These cells were grown at 28 °C with 5% CO_2_.

### 2.2. Dengue NS1 ELISA-Positive Human Serum Samples

Serum samples (*n* = 4) from dengue fever patients were collected with proper information and prior written patient consent. Serum samples from a few apparently healthy individuals were also collected, to serve as negative controls. The study was conducted according to the guidelines of the Declaration of Helsinki and approved by the respective Institutional Ethical Committees of Calcutta National Medical College and CSIR-IICB, Kolkata (certificate numbers *IICB/human ethics/07.09.2015/dengue* and *IICB/human ethics/02.02.2016/dengue*). All serum samples were primarily confirmed as dengue virus-positive or negative by NS1-ELISA test (Platelia, Biorad).

### 2.3. Virus Culture

Our earlier study has indicated the high prevalence of dengue virus in both the adult and the larva form of *Aedes aegypti* mosquitoes [14]. Therefore, mosquitoes were collected for dengue virus isolation from them. A pool of *Aedes aegypti* mosquitoes was selected, homogenized, and centrifuged. The supernatant was collected and filtered using Millipore 0.22 µM PES syringe filters. Semi-confluent (70%) monolayer of C6/36 cells was infected with filtered solution and adsorption was performed for 2 h under normal cell culture conditions, with intermittent shaking every 15 min.

Cells with virus were incubated for 120 h. Three such passages were given in C6/36. During harvesting, T-25 flasks (NUNC, Hamburg, Germany), containing cells, were scraped in 1 mL supernatant. The cells were freeze-thawed and centrifuged at 13,000 rpm for 15 min at 4 °C. The supernatant was aliquoted and stored at −80 °C as virus stocks. Virus serotyping was performed, as described earlier [14]. Virus titer for the C6/36 grown stock of DENV1 and the NS1-positive human serum samples was determined using SYBR-based, one-step qRT-PCR with Luna Universal One-Step qRT-PCR reagent (NEB). Quantstudio 5 (AB) was used to run the qPCRs. Primers as described by Lanciotti were used in qRT-PCRs [12].

### 2.4. Sequence Selection for Designing Molecular Beacon

**Scheme 2:**
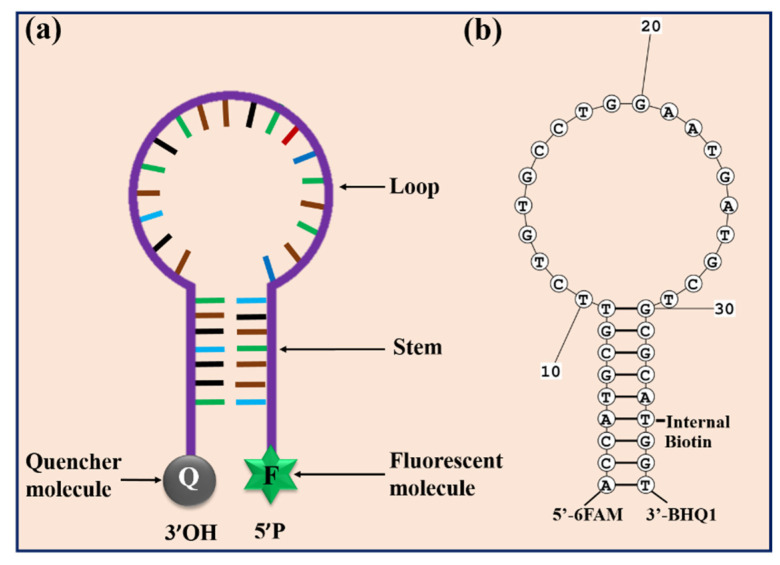
Diagram of a typical molecular beacon (**a**) and the design of pan-dengue molecular beacon used in this study (**b**).

A pan-dengue target sequence (PDC) was selected, based on a conserved region of DENV genome. A complementary sequence was incorporated in the probe (pDENV). The sequence of the probes, with both the pan-dengue and DENV2-specific sequence, has been checked for loop formation using the RNAstructure webserver [15]. The PDSB probe forms a perfect MB (shown in Scheme 2b) and was exclusively studied in this work for the detection of pan-dengue (PDC) sequence. The DENV2-specific D2C target showed three base-pairings at the lowest energy, as predicted by RNAstructure. As a result, the molecular beacon structure of D2P probe, being complementary to D2C target also has three base-pairings and is not a perfect beacon (Figure S1, Electronic Supporting Information, ESI). We used the D2C target for checking cross-reactivity with the pan-DENV probe.

The sequence and designing of biotinylated molecular beacon (PDSB/PDTG), used in this study for detection of pan-dengue RNA, is as follows:

**PDTG:** 5′-6FAM-ACCATGCG (TTCTGTGCCTGGAATGATGCTG) CGCA (Internal BIOTIN-dT)-GGT-BHQ1-3′

**PDSB:** This probe has the same sequence as PDTG, but it is not biotinylated.

**PDC:** Pan-dengue target sequence: 5′-CAGCATCATTCCAGGCACAGAA-3′

The DENV2-specific probe (D2P) used for detection of the DENV2-specific target (D2C) had the following sequence.

**D2P**: 5′-6FAM-ACCATGCG (CGCCACAAGGGCCATGAACAG) CGCATGGT-BHQ1-3′

**D2C**: 5′-CTGTTCATGGCCCTTGTGGCG-3′

The sequences in the parenthesis in the above two probes represent the DENV complementary/capture sequence.

All probes and synthetic target sequences were procured from IDT, Germany.

### 2.5. Fluorophore-Quencher Selection

In the literature, several fluorophores and quenchers are reported. The fluorophore absorbs light energy of a specific wavelength and re-emits light at a longer wavelength. The fluorophores are characterised by their specific excitation and emission range, molar absorption coefficient, quantum yield, lifetime, Stokes shift, etc. Based on their characteristics, and after consulting the available literature [16,17], FAM was selected as the fluorophore for our experiments. Quenching is a process that decreases the fluorescence intensity of a given substance. Several quenchers are available and, based on their quenching properties with a given fluorophore, the fluorophore-quencher combination was selected. The specific quencher selected for our study was BHQ-1 [16,17].

### 2.6. Molecular Beacon Designing

Molecular beacons are hairpin-shaped structures, along with a fluorophore and quencher molecules. The fluorescence in the quenched fluorophore is restored when there is binding to a target nucleic acid sequence in the loop region. This is a non-radioactive method for detecting specific sequences of nucleic acids. A typical molecular beacon has four parts: a loop, stem region, 5′fluorophore, and 3′quencher (Scheme 2).

The molecular beacon used in the experiments consisted of the pan-dengue complementary sequence at the loop region. The stem length plays a crucial role, and the exact length was selected from three different stem lengths tested. The fluorophore-quencher combination was selected, as discussed above. These probes are biotinylated in the stem region, so that they can be immobilized to the streptavidin-coated magnetic beads used for the separation and concentration of target RNA from the serum.

### 2.7. Standardization of Different Parameters in Fluorometer

All the fluorescence measurements were taken in the photon technology international (PTI) quanta master spectrofluorometer (QM-40), which is equipped with Peltier for controlling the temperature. Different parameters of the fluorometer, such as excitation and emission slit width, voltage, excitation and emission wavelength, etc., were optimized, along with the volume of mixture, incubation temperature, and time; the following standardised parameters were used for all the measurements:

Excitation slit width: 1.25 mm
Emission slit Width: 1.25 mm
Voltage: 121 V
Integration time: 0.1 s
Excitation wavelength: 495 nm
Emission range: 505–650 nm
Maximum emission wavelength: 519 nm
Volume of the mixture: 20 µL
Incubation temperature: 50 °C
Incubation time: 15 min

### 2.8. Molecular Beacon Experiments

The molecular beacon (PDSB), designed and selected for pan-dengue detection, was tested with its target probe (PDC). At this stage, the standardizations were performed for the buffer, exact temperature, and incubation time. The fluorometer settings were also standardized. For the standardization experiments, higher dilutions of the target sequence were used. The D2P probe, designed for detecting DENV2-specific target (D2C), was also used for initial standardization experiments. All the measurements were taken in three replicates.

#### 2.8.1. Checking for the Sensitivity

To test the sensitivity, the target sequence was diluted, until 10^2^ copies were mixed with PDSB and incubated for 15 min at 50 °C. Thereafter, the fluorescence intensity of all mixtures was measured by fluorometer.

#### 2.8.2. Checking for the Specificity

The specificity of the molecular beacon was tested using a different target sequence, instead of the pan-dengue target sequence. D2C is the DENV2-specific target sequence and PDSB probe is specific for detecting pan-dengue target sequence. Hence, D2C, which is non-specific for the PDSB probe, was used to test the specificity of the PDSB probe. Different concentrations of D2C target were mixed with PDSB, incubated at 50 °C for 15 min, and the fluorescence intensity was measured.

#### 2.8.3. Testing of Viral RNA as a Target

To test whether the viral RNA can be directly detected by the PDSB probe, RNA was isolated from the DENV1-infected C6/36 cell culture. The copy number was determined by quantitative RT-PCR and different dilutions were made accordingly.

### 2.9. Experiments with Biotinylated Molecular Beacons

The above-mentioned molecular beacon (PDSB) was tagged with biotin (PDTG). This biotinylated beacon was immobilised on streptavidin-coated magnetic beads for the detection of viral RNA in the serum samples. The tagged molecular beacons were checked using the pan-dengue target sequence (PDC). All the other parameters remained the same.

### 2.10. Immobilization of the Molecular Beacons with the Magnetic Beads

The streptavidin-coated magnetic beads (GeneScript, NJ, USA, Cat. No. L00424) were prepared, following the manufacturer’s instructions. A total of 1 mL of settled beads can bind to ~50 nmol of biotinylated oligonucleotides. Accordingly, PDTG was mixed with Strep-MagBeads, and three washings with 1X PBS were performed, in order to remove any unbound probes, using the magnetic separation rack. This conjugated combination of Strep-Mag Beads and biotinylated pan-dengue PDTG probe (Mg-PDTG) was used for further measurements.

### 2.11. Testing Viral RNA with the Conjugated Molecular Beacons and Magnetic Beads

RNA was isolated from C6/36 cell culture grown DENV1 stock (originally collected from the pooled mosquito samples), as well as from the human serum samples, as described previously [12]. The extracted RNA from DENV1 infected cells was serially diluted in fetal bovine serum (FBS) (Sigma). The conjugated molecular beacons (Mg-PDTG) were added to them. The mixture was incubated at room temperature for 1 h. The conjugated beads were separated from the serum using the magnetic racks. They were washed three times in TES and resuspended in the TES buffer. The measurements were taken, as described earlier.

### 2.12. Detection of Viral RNA by Processing the Virus In Situ

A C6/36 grown DENV1 virus stock with known copy number was processed and serially diluted in FBS. Mg-PDTG beads were added to it. They were processed following the protocol described below. The measurements were taken, following the standardised protocol.

### 2.13. Detailed Protocol for the Detection of Dengue Virus in Serum Samples Directly

A 2X volume (for example, 100 µL) of streptavidin-tagged magnetic beads was mixed with 1X volume (for example, 50 µL) of biotinylated molecular beacons in a 2 mL tube, and the tube was inverted gently 3–4 times for proper mixing. The tube was then incubated at room temperature with mixing (on a shaker or rotor) for one hour. The beads were collected using the magnetic separation rack and the supernatant was discarded. A total of 500 µL of TES buffer was added to the tube and mixed well. The magnetic separation rack was again used to collect the beads and the supernatant was discarded. Wash steps were repeated three times and the beads were resuspended in 240 µL TES buffer. The mixture was kept at 4 °C for further use.

200 µL serum (from infected patients or FBS containing known DENV1 dilutions) was taken in a 2 mL tube. The sample was sonicated for three times with 10 s pulse under chilled condition. In another tube, 200 µL of FBS was taken as a negative control. 10 µL of the bead mix was added to 200 µL of each serum. The samples were incubated by rotating in rotor for 30 min with 20 rpm. The tubes were briefly centrifuged at 4000 rpm. The mixture was then washed following the method described above for the washing of beads. After the third wash, beads were resuspended in 100 µL TES. 20 µL of this mixture was used for taking the measurement. The mixture was then incubated at 50 °C for 15 min inside the fluorometer. The measurement of fluorescence was taken thereafter. Three measurements were taken for each sample with the optimized parameters set in the fluorometer.

The negative control with FBS served as the background fluorescence, which was deducted from all the other readings to come to the conclusion. Any measurement above the background was considered as DENV positive signal. 10^7^ copies of PDC were used as positive control. Four NS1-ELISA positive and three NS1-ELISA negative human serum samples were used to test the method at pilot level.

## 3. Results

### 3.1. Standardization of Molecular Beacon

**Figure 1:**
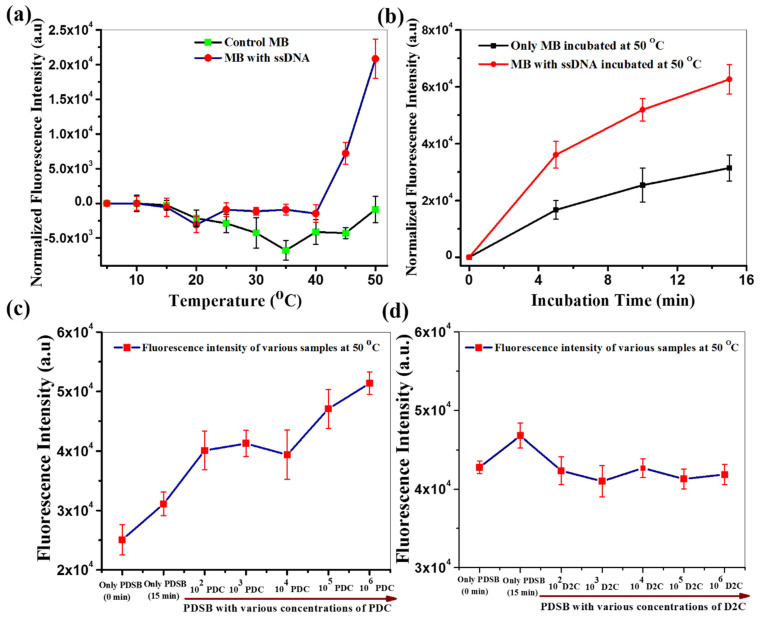
(**a**–**d**) Molecular beacon (MB) standardization (**a**) comparison of fluorescence intensity of molecular probe, in absence and presence of ssDNA with increasing temperature; (**b**) Relative fluorescence increment of MB and MB with ssDNA when incubated for different time periods at 50 °C; (**c**) PDSB probe was incubated with different concentrations of PDC target at 50 °C to check the sensitivity of PDSB probe; (**d**) PDSB probe was incubated with different concentrations of non-specific target (D2C) to check for the specificity of PDSB probe. Each experiment was performed thrice (*n* = 3), and error bars represent the standard deviation of fluorescence intensity measurement from average value.

In principle, molecular beacon should not give fluorescence alone, as the quencher and fluorophore molecule stay in close proximity, but in the presence of the target nucleic acid sequence, it gives fluorescence. Hence, to test the activity of the designed probe, it was incubated in presence and absence of target sequence (ssDNA, such as PDC) and the temperature of the mixtures was also raised. It was observed that the probe was showing fluorescence at 45 °C, and the maximum difference of fluorescence intensity between only probe and probe with ssDNA was observed at 50 °C (Figure 1a; Figures S2 and S3, ESI). We had also tried higher temperatures (45–75 °C) with a different probe (D2P) and target sequence (D2C), in order to measure the optimum temperature for the detection of target. We found maximum fluorescence difference at 50 °C (Figure S4, ESI).

Along with this, the probe was incubated with ssDNA for different time period, and it was found that, when the mixture (probe with ssDNA) was incubated for 15 min, there was maximum difference between control probe and probe with ssDNA (Figure 1b; Figure S5, ESI). Hence, the incubation temperature and time for all the experiments performed with molecular beacon in this work were fixed at 50 °C and 15 min, respectively. The detailed parameters used to measure the fluorescence and overall workflow of the current detection process are mentioned in the methods section (Figures S6 and S7; ESI).

#### 3.1.1. Checking for the Sensitivity

To test the sensitivity of PDSB, the target sequence (PDC) was diluted till 10^2^ copies. This method was able to detect up to 10^2^ copies of the target present in the solution. The level of fluorescence intensity was consistent at the higher level of 10^4^ to 10^6^ copies. At the level of 10^2^ or 10^3^ copies, the fluorescence intensity did not increase proportionately; nonetheless, the fluorescence level was higher than the control (Figure 1c; Figure S8, ESI).

#### 3.1.2. Checking for the Specificity

The specificity of the molecular beacons was tested using a different target sequence instead of the pan-dengue target sequence. It was observed that, in the presence of non-specific target, there was no increase in the fluorescence level. For example, D2C sequence, which is a non-specific target for the PDSB probe, was used to test the specificity of the PDSB probe. There was no increment of fluorescence intensity, up to 10^6^ copies of D2C (Figure 1d; Figure S9, ESI).

#### 3.1.3. Testing of Viral RNA as a Target Using Molecular Beacon Only

Viral RNA was isolated from the DENV1 infected C6/36 cell culture. The copy number was measured by quantitative RT-PCR and the RNA dilutions were made. These samples were checked for binding to the PDSB probe, using the same protocol, but no change in the fluorescence level was detected. So, it was concluded that direct and isolated RNA would not bind with molecular beacon (Figure S10, ESI).

### 3.2. Experiments with Biotinylated Molecular Beacons

**Figure 2:**
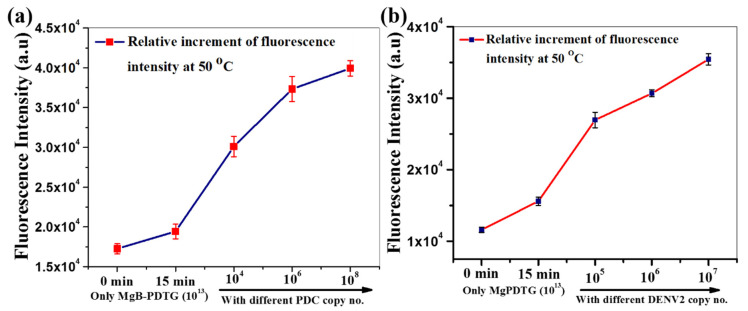
Evaluation of the sensitivity of the probes. (**a**) Relative fluorescence increment of only biotin-tagged, pan-dengue probe (PDTG) and PDTG with a different copy numbers of target PDC, (**b**) Strep-MagBeads, and biotinylated pan-dengue probe (Mg-PDTG) only, as well as in the presence of DENV1 target sequence at 50 °C. Each experiment was performed thrice (*n* = 3), and error bars represent the standard deviation of fluorescence intensity measurement from average value.

The PDSB molecular beacon was tagged with biotin (PDTG). This biotinylated beacon was conjugated to the streptavidin-coated magnetic beads for the detection of virus directly in the serum samples. The tagged molecular beacons were initially checked using pan-dengue target sequence (PDC). All the other parameters remained the same. It has been observed that the results remained unchanged after the biotin conjugation (Figure 2a; Figure S11, ESI).

#### 3.2.1. Testing Viral RNA with the Conjugated Molecular Beacons and Magnetic Beads

Isolated viral RNA was serially diluted in FBS. The conjugated molecular beacon (Mg-PDTG) was added to the RNA. The mixture was incubated at room temperature for 1 h. The conjugated beads were separated from the serum using the magnetic racks. They were washed three times and resuspended in the buffer. The measurements were taken as described earlier. There was no difference in the fluorescence level, compared to the control, again suggesting that direct and isolated viral RNA could not be detected.

#### 3.2.2. Detection of Viral RNA by Processing the Virus In Situ

The measurements were taken using serially diluted C6/36 grown DENV1 virus stock with known copy number following the standardised protocol (Figure S7, ESI). The Mg-PDTG probe detected DENV1 at all test concentrations, starting from 10^5^ to 10^7^ copies (Figure 2b; Figure S12, ESI). The fluorescent level increased with increased copy numbers, such as 10^6^ and 10^7^.

### 3.3. Clinical Samples Testing

**Figure 3:**
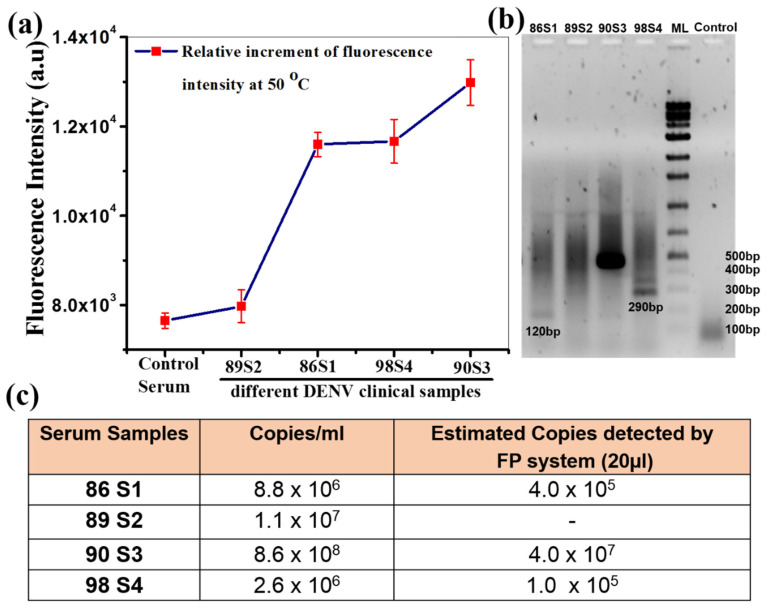
(**a**–**c**) Detection of clinical DENV serum samples. (**a**) Relative increment of fluorescence intensity of various clinical samples, measured with Mg-PDTG probe. (**b**) Agarose gel (1%) electrophoresis image of the qRT-PCR products, obtained from viral RNA isolated from different clinical DENV samples. (**c**) Analysis of qRT-PCR data to measure the DENV copies/mL in different clinical samples. FP system: fluorescence probe system. Each experiment was performed thrice (*n* = 3), and error bars represent the standard deviation of fluorescence intensity measurement from the average value.

Four dengue NS1-ELISA positive (86S1, 89S2, 90S3, and 98S4) and three NS1-ELISA negative human serum samples (S65, S66, and S67) were tested by our method. The fluorescence-based assay, with the Mg-PDTG probe, was performed to see the activity of this probe in the presence of real dengue samples. It was found that the Mg-PDTG probe could efficiently detect dengue in serum samples (Figure 3a; Figure S13, ESI). These serum samples were further confirmed as DENV-positive by qRT-PCR on RNA, isolated from these samples (Figure 3b). DENV copy numbers, in these four samples, were also determined by qRT-PCR (Figure 3c). The serum samples that were NS1-ELISA negative (S65, S66, and S67) produced background level fluorescence, which was much lower, compared to the positive control. They were also found DENV RT-PCR negative (Figure S14, ESI).

## 4. Discussion

This is a rapid and easy-to-use method for the detection of dengue virus from the infected patients’ serum samples, with the potential to be improvised to detect different dengue serotypes, as well as other viruses. It is superior to the NS1-ELISA-based method of detection, as it is based on the detection of nucleic acid, which gives more precise results. The cross-reactivity of antibody responses among co-circulating flaviviruses is a confounding issue in providing a differential diagnosis by ELISA. This is particularly true in the context of the Indian subcontinent, where different dengue serotypes, Japanese encephalitis and Zika viruses, are co-circulating [18,19,20]. The ongoing pandemic, due to SARS-CoV-2, has also complicated the diagnosis of DENV, as there are reports of false positivity and cross-reactivity between these two viruses [3,4,5,6]. It has been well-documented that the NS1-ELISA method, for early dengue detection, though very specific, is rather less sensitive, with a wide variation ranging from 52–86% sensitivity in different studies using various commercially available kits in different geographical locations [21,22,23,24,25,26,27]. The diagnostic accuracy of NS1-ELISA has also been analyzed by meta-analysis and it has, again, been confirmed that NS1-ELISA lacks sensitivity [28,29]. It has also been reported that the sensitivity differs towards specific serotypes; for example, NS1-ELISA was unable to detect DENV4 in Brazil, resulting in underreporting of this serotype [30]. This low sensitivity, therefore, leaves a large window of infections being missed as false-negatives, possibly due to variability of NS1 protein sequences among rapidly evolving DENV strains, which could not be captured by the antibodies used in the ELISA kits. We observed that our system can detect DENV1 from 10^2^ copies, but linearity was only perceptible from 10^4^ copies and higher. In this respect, qRT-PCR certainly has a higher sensitivity, as the detection limit is even less than 10 copies [10,11,12,13]. Our method is quicker and easy-to-perform, compared to qPCR, as it does not need RNA extraction and PCR-amplification but still provides nucleic-acid-based specific detection. A comparative analysis of the above three methods of early dengue diagnosis has been tabulated (Table S1, ESI).

The probes, described in this study, can be easily modified by changing the DENV complementary sequence in the loop region to cover the genetic variability of the circulating strains. This is far easier and faster than modifying ELISA kits to adapt to the changing DENV strains. This method can also be further improvised, in order to detect different DENV serotypes. This differential diagnosis is not presently possible using the currently used ELISA kits. This method is superior to the RT-PCR method of nucleic acid detection, as it bypasses the need for time-consuming and labour-intensive processes of isolating nucleic acid from serum, performing RT-PCR, and observing the bands after running them in agarose gel electrophoresis. By this method, dengue viruses, with copy numbers as low as 10^2^, were consistently detected. In the case of the clinical sample, 89S2, no clear PCR band was visible in the agarose gel with the qRT-PCR products; hence, the copy numbers measured from qRT-PCR might have come from the non-specific binding, seen as a smear in the gel. The same sample didn’t give any detectable fluorescence, indicating that the sample, indeed, had a lower level of viral RNA, undetectable by both the qRT-PCR and our molecular beacon-based method. In case of active infection, it has been documented that around 10^4^ to 10^6^ RNA copy numbers are present per mL of serum [31]. Therefore, this method can easily detect an ongoing, active infection with precision. The proof-of-principle of this method can be utilized to develop a hand-held system that can be used as a point-of-care device for ready bedside detection of pathogens. A multi-centric study for the clinical validation of a large number of patients’ serum samples is warranted.

## 5. Patents

The work has been provisionally e-filed for patenting the method in India on 5 May 2020 (Ref. No. CSIR-IPU Cell: 0166NF2019; Dated: 3 October 2019).

## Data Availability

Data is contained within the article and Supplementary Materials.

## Supplementary Materials

The following are available online at https://www.mdpi.com/article/10.3390/bios11120479/s1, Figure S1: RNAstructure webserver-predicted structure of DENV2-specific target D2C (a) and the DENV2-specific target probe D2P (b) used for detection of D2C; Figures S2, S3: Molecular beacon (MB) standardization by measuring fluorescence; Figures S4, S5: Fluorescence intensity measurement of only D2P probe, D2P with target D2C, only MB and MB with ssDNA; Figure S6: Table showing fluorometer parameters used to measure the fluorescence intensity; Figure S7: Schematic view of the overall process; Figures S8, S9, S10: Fluorescence intensity measurement of PDSB probe only and PDSB probe with different concentrations of PDC, D2C, and isolated DENV1 RNA; Figure S11: Fluorescence measurement of only biotin tagged pan-dengue probe (PDTG) and PDTG with different copy numbers of target PDC; Figure S12: Fluorescence intensity measurement of biotinylated pan-dengue probe immobilized on Strep-MagBeads (Mg-PDTG) only and in presence of DENV1; Figure S13: Detection of clinical DENV samples by measuring fluorescence (using Mg-PDTG); Figure S14: Detection of DENV-negative clinical samples (using Mg-PDTG); Table S1: Comparison between NS1-ELISA, qRT-PCR and molecular-beacon-based dengue diagnosis.

## References

[1] WHO Improving Data for Dengue. https://www.who.int/activities/improving-data-for-dengue.

[2] Bhatt S., Gething P.W., Brady O.J., Messina J.P., Farlow A.W., Moyes C.L., Drake J.M., Brownstein J.S., Hoen A.G., Sankoh O. (2013). The global distribution and burden of dengue. Nature.

[3] Nath H., Mallick A., Roy S., Sukla S., Biswas S. (2021). Computational modelling supports that dengue virus envelope antibodies can bind to SARS-CoV-2 receptor binding sites: Is pre-exposure to dengue virus protective against COVID-19 severity?. Comput. Struct. Biotechnol. J..

[4] Lustig Y., Keler S., Kolodny R., Ben-Tal N., Atias-Varon D., Shlush E., Gerlic M., Munitz A., Doolman R., Asraf K. (2021). Potential Antigenic cross-reactivity between severe acute respiratory syndrome coronavirus 2 (SARS-CoV-2) and dengue viruses. Clin. Infect. Dis..

[5] Yan G., Lee C.K., Lam L.T.M., Yan B., Chua Y.X., Lim A.Y.N., Phang K.F., Kew G.S., Teng H., Ngai C.H. (2020). Covert COVID-19 and false-positive dengue serology in Singapore. Lancet Infect. Dis..

[6] Nath H., Mallick A., Roy S., Sukla S., Basu K. (2020). Dengue antibodies can cross-react with SARS-CoV-2 and vice versa-Antibody detection kits can give false-positive results for both viruses in regions where both COVID-19 and Dengue co-exist. MedRxiv.

[7] Kembuan G.J. (2020). Dengue serology in Indonesian COVID-19 patients: Coinfection or serological overlap?. IDCases.

[8] Bicudo N., Bicudo E., Costa J.D., Castro J.A.L.P., Barra G.B. (2020). Co-infection of SARS-CoV-2 and dengue virus: A clinical challenge. Braz. J. Infect. Dis..

[9] Butt M.H., Ahmad A., Misbah S., Mallhi T.H., Khan Y.H. (2020). Dengue fever and COVID-19 coinfection; a threat to public health for coepidemic in Pakistan. J. Med. Virol..

[10] Parkash O., Shueb R.H. (2015). Diagnosis of dengue infection using conventional and biosensor based techniques. Viruses.

[11] Muller D.A., Depelsenaire A.C., Young P.R. (2017). Clinical and laboratory diagnosis of dengue virus infection. J. Infect. Dis..

[12] Lanciotti R.S., Calisher C.H., Gubler D.J., Chang G.J., Vorndam A.V. (1992). Rapid detection and typing of dengue viruses from clinical samples by using reverse transcriptase-polymerase chain reaction. J. Clin. Microbiol..

[13] Waggoner J.J., Abeynayake J., Sahoo M.K., Gresh L., Tellez Y., Gonzalez K., Ballesteros G., Pierro A.M., Gaibani P., Guo F.P. (2013). Single-reaction, multiplex, real-time RT-PCR for the detection, quantitation, and serotyping of dengue viruses. PLoS Negl. Trop. Dis..

[14] Sukla S., Ghosh A., Saha R., De A., Adhya S., Biswas S. (2018). In-depth molecular analysis of a small cohort of human and Aedes mosquito (adults and larvae) samples from Kolkata revealed absence of Zika but high prevalence of dengue virus. J. Med. Microbiol..

[15] Reuter J.S., Mathews D.H. (2010). RNAstructure: Software for RNA secondary structure prediction and analysis. BMC Bioinform..

[16] Marras S.A.E. (2008). Interactive fluorophore and quencher pairs for labeling fluorescent nucleic acid hybridization probes. Mol. Biotechnol..

[17] Marras S.A.E. (2006). Selection of fluorophore and quencher pairs for fluorescent nucleic acid hybridization probes. Methods Mol. Biol..

[18] Mukherjee S., Dutta S.K., Sengupta S., Tripathi A. (2017). Evidence of dengue and chikungunya virus co-infection and circulation of multiple dengue serotypes in a recent Indian outbreak. Eur. J. Clin. Microbiol. Infect. Dis..

[19] Kulkarni R., Sapkal G.N., Kaushal H., Mourya D.T. (2018). Japanese Encephalitis: A brief review on Indian perspectives. Open Virol. J..

[20] Yadav P.D., Malhotra B., Sapkal G., Nyayanit D.A., Deshpande G., Gupta N., Padinjaremattathil U.T., Sharma H., Sahay R.R., Sharma P. (2019). Zika virus outbreak in Rajasthan, India in 2018 was caused by a virus endemic to Asia. Infect. Genet. Evol..

[21] Solanke V.N., Karmarkar M.G., Mehta P.R. (2015). Early dengue diagnosis: Role of rapid NS1 antigen, NS1 early ELISA, and PCR assay. Trop. J. Med. Res..

[22] Ambrose J.H., Sekaran S.D., Azizan A. (2017). Dengue Virus NS1 Protein as a Diagnostic Marker: Commercially Available ELISA and Comparison to qRT-PCR and Serological Diagnostic Assays Currently Used by the State of Florida. J. Trop. Med..

[23] Hermann L.L., Thaisomboonsuk B., Poolpanichupatam Y., Jarman R.G., Kalayanarooj S., Nisalak A., Yoon I.-K., Fernandez S. (2014). Evaluation of a dengue NS1 antigen detection assay sensitivity and specificity for the diagnosis of acute dengue virus infection. PLoS Negl. Trop. Dis..

[24] Hunsperger E.A., Yoksan S., Buchy P., Nguyen V.C., Sekaran S.D., Enria D.A., Vazquez S., Cartozian E., Pelegrino J.L., Artsob H. (2014). Evaluation of commercially available diagnostic tests for the detection of dengue virus NS1 antigen and anti-dengue virus IgM antibody. PLoS Negl. Trop. Dis..

[25] Aryati A., Trimarsanto H., Yohan B., Wardhani P., Fahri S., Sasmono R.T. (2013). Performance of commercial dengue NS1 ELISA and molecular analysis of NS1 gene of dengue viruses obtained during surveillance in Indonesia. BMC Infect. Dis..

[26] Lima M.D.R.Q., Nogueira R.M.R., Schatzmayr H.G., dos Santos F.B. (2010). Comparison of three commercially available dengue NS1 antigen capture assays for acute diagnosis of dengue in Brazil. PLoS Negl. Trop. Dis..

[27] Guzman M.G., Jaenisch T., Gaczkowski R., Ty Hang V.T., Sekaran S.D., Kroeger A., Vazquez S., Ruiz D., Martinez E., Mercado J.C. (2010). Multi-country evaluation of the sensitivity and specificity of two commercially-available NS1 ELISA assays for dengue diagnosis. PLoS Negl. Trop. Dis..

[28] Shan X., Wang X., Yuan Q., Zheng Y., Zhang H., Wu Y., Yang J. (2015). Evaluation of the diagnostic accuracy of nonstructural pro-tein 1 Ag-based tests for dengue virus in Asian population: A meta-analysis. BMC Infect. Dis..

[29] Da Costa V.G., Marques-Silva A.C., Moreli M.L. (2014). A meta-analysis of the diagnostic accuracy of two commercial NS1 antigen ELISA tests for early dengue virus detection. PLoS ONE.

[30] Sea V.R., Cruz A.C., Gurgel R.Q., Nunes B.T., Silva E.V., Dolabella S.S., dos Santos R.L. (2013). Underreporting of dengue-4 in Brazil due to low sensitivity of the NS1 Ag test in routine control programs. PLoS ONE.

[31] Wang W.K., Sung T.L., Tsai Y.C., Kao C.L., Chang S.M., King C.C. (2002). Detection of dengue virus replication in peripheral blood mononuclear cells from dengue virus type 2-infected patients by a reverse transcription-real-time PCR assay. J. Clin. Microbiol..

