# Supplementary material for: A Rapid and Easy-to-Perform Method of Nucleic-Acid Based Dengue Virus Diagnosis Using Fluorescence-Based Molecular Beacons
Source: Biosensors (Basel). 2021 Nov 26;11(12):479. doi: 10.3390/bios11120479 (PMC8699591; doi:10.3390/bios11120479)
Supplement: Supplementary file 1 [file biosensors-11-00479-s001.zip › biosensors-1404561-Supplementary.pdf]

biosensors

MDPI

Supporting Information

A Rapid and Easy-to-Perform Method of Nucleic-Acid Based Dengue Virus Diagnosis Using Fluorescence-Based Molecular Beacons

Soumi Sukla [1,2], Prasenjit Mondal [1,3,4], Subhajit Biswas [1,4,*] and Surajit Ghosh [1,4,5,*]

1 CSIR-Indian Institute of Chemical Biology, 4, Raja S.C. Mullick Road, Kolkata 700032, West Bengal, India; soumi.sukla@niperkolkata.edu.in (S.S.); pmondal@mgh.harvard.edu (P.M.)
2 National Institute of Pharmaceuticals Education and Research, 168, Maniktala Main Road, Kolkata 700054, West Bengal, India
3 Department of Neurology, Massachusetts General Hospital, Harvard Medical School, Charlestown, MA 02129, USA
4 Academy of Scientific and Innovative Research (AcSIR), Ghaziabad 201002, Uttar Pradesh, India
5 Department of Bioscience & Bioengineering, Indian Institute of Technology Jodhpur, NH 62, Surpura Bypass Road, Karwar 342037, Rajasthan, India
* Correspondence: subhajit.biswas@iicb.res.in (S.B.); sghosh@iitj.ac.in (S.G.)

Citation: Sukla, S.; Mondal, P.; Biswas, S.; Ghosh, S. A Rapid and Easy-to-Perform Method of Nucleic-Acid Based Dengue Virus Diagnosis Using Fluorescence-Based Molecular Beacons. *Biosensors* **2021**, *11*, 479. https://doi.org/10.3390/bios11120479

Received: 16 September 2021
Accepted: 19 November 2021
Published: 26 November 2021

Publisher's Note: MDPI stays neutral with regard to jurisdictional claims in published maps and institutional affiliations.

Copyright: © 2021 by the authors. Submitted for possible open access publication under the terms and conditions of the Creative Commons Attribution (CC BY) license (https://creativecommons.org/licenses/by/4.0/).

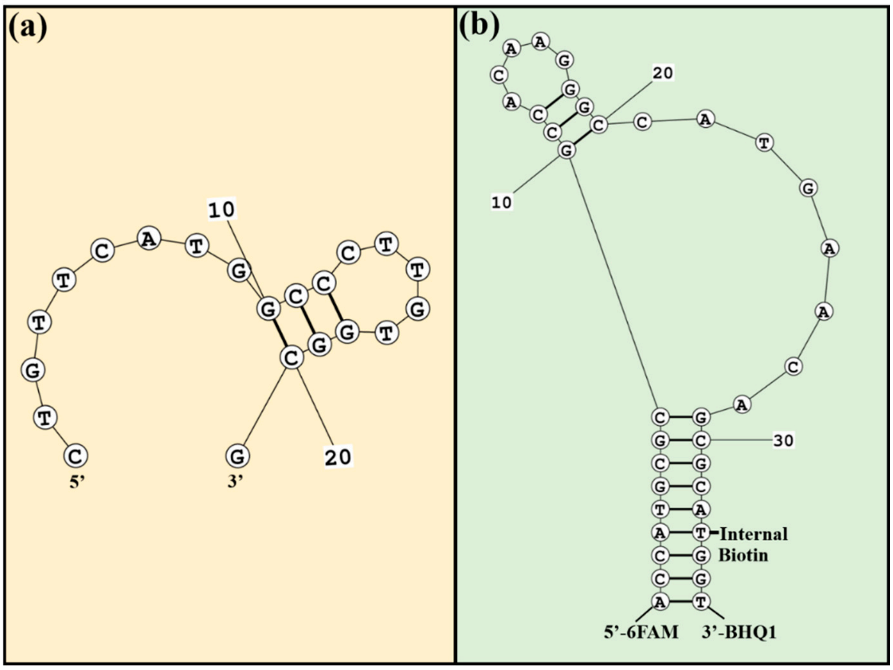

Figure S1. RNAstructure webserver-predicted structure of DENV2-specific target D2C (a) and the DENV2-specific target probe D2P (b) used for detection of D2C. This same D2C target was also used for checking cross-reactivity with the pan-DENV probe.

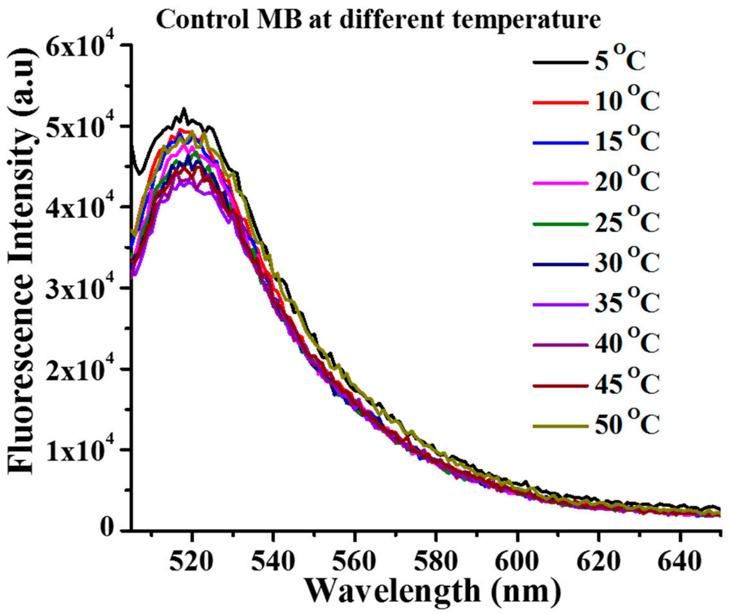

Figure S2. Molecular beacon (MB) standardization, fluorescence intensity of control molecular beacon with increasing temperatures.

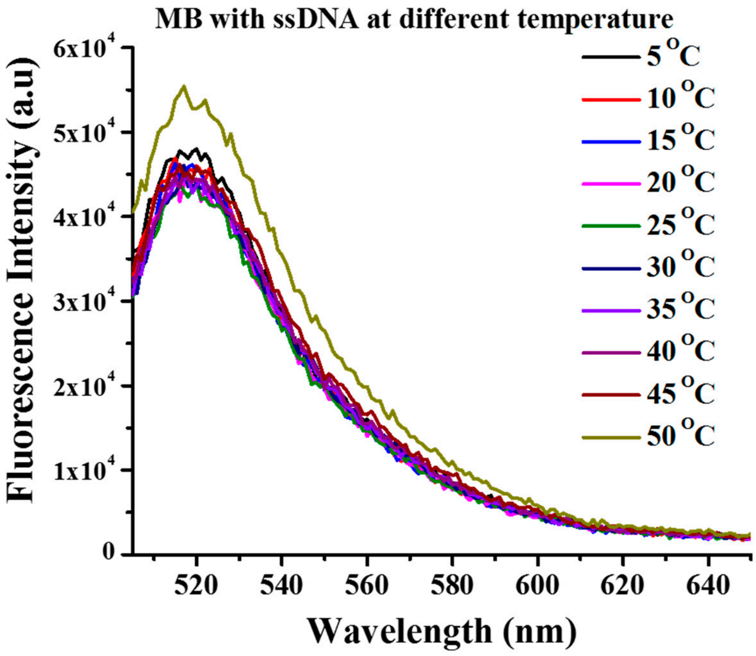

Figure S3. Molecular beacon (MB) standardization, fluorescence intensity of control molecular beacon with target ssDNA with increasing temperatures.

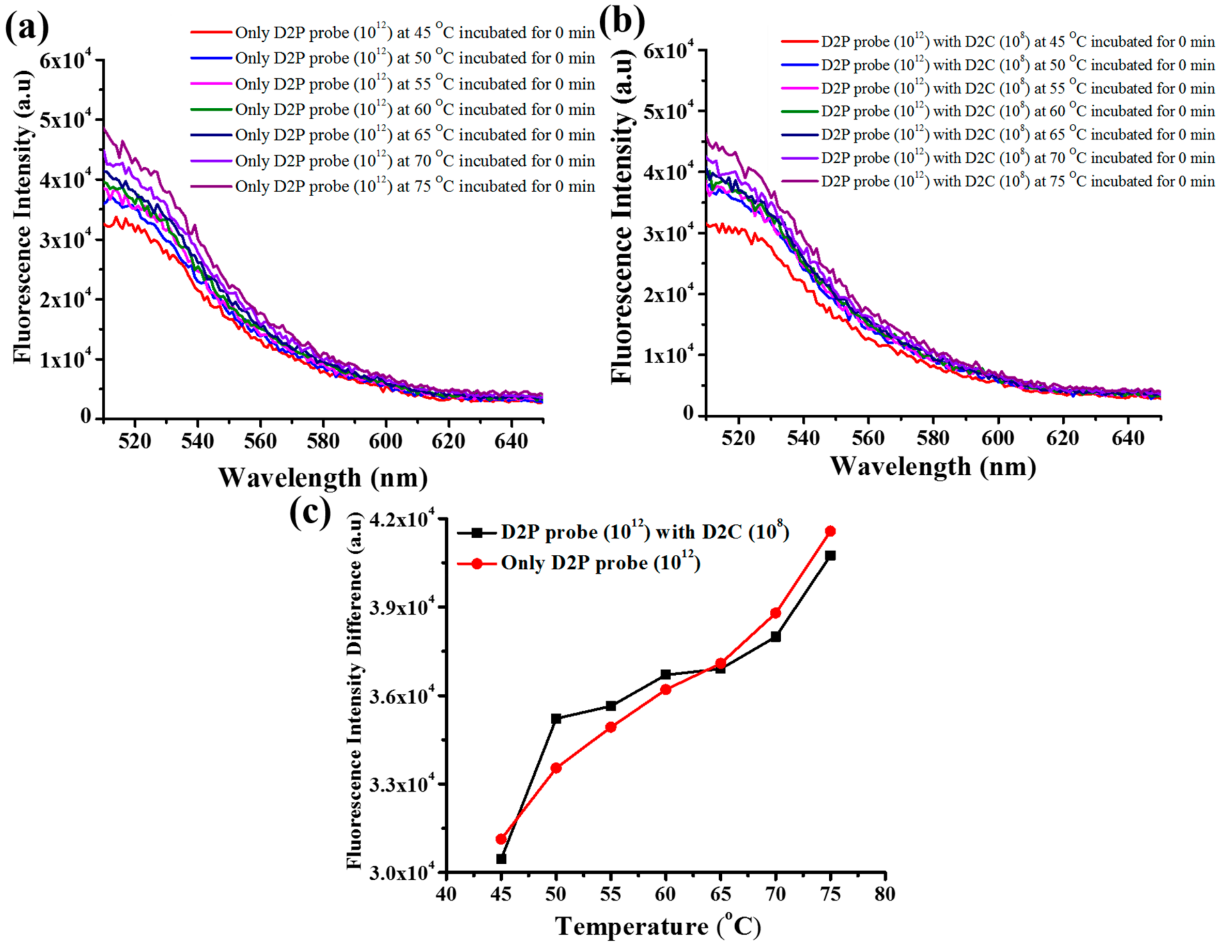

Figure S4. Fluorescence intensity measurement of only D2P probe (a), D2P probe with D2C target sequence (b) with increasing temperature from 45 ^{0}C to 75 ^{0}C. (c) Relative fluorescence increment upon adding D2C target sequence to D2P probe at different temperatures. Maximum fluorescence difference was observed at 50 ^{0}C.

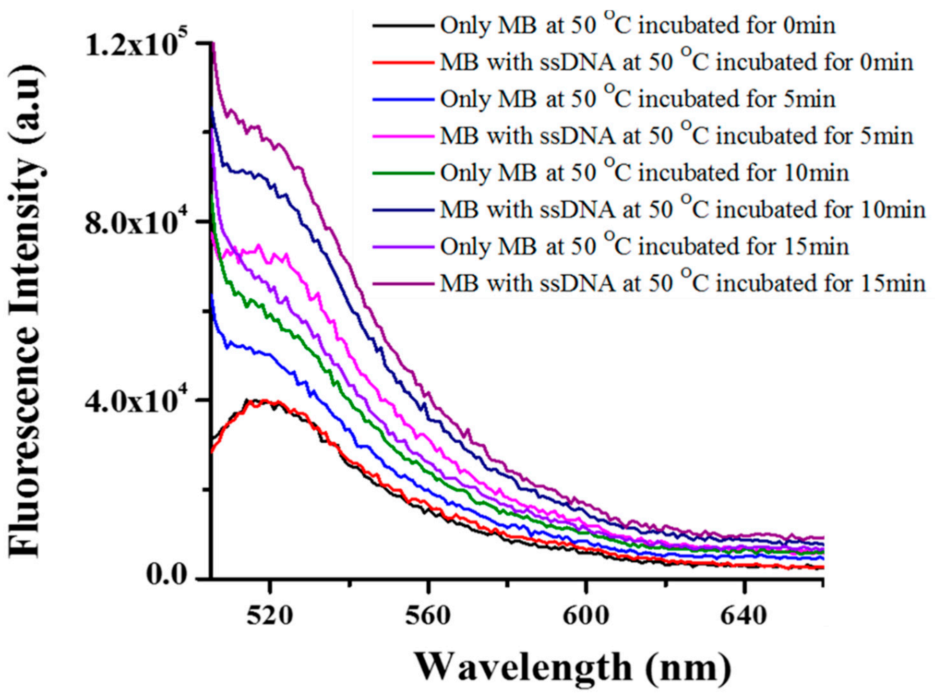

Figure S5. Fluorescence intensity measurement of MB and MB with ssDNA after incubating them for different time periods at 50 ⁰C.

Fluorometer Settings	Values
Excitation Slit width	1.25 mm
Emission Slit Width	1.25 mm
Voltage	121v
Integration time	0.1sec
Excitation Wavelength	495 nm
Measured Emission range	505-650 nm
Maximum Emission wavelength	519 nm

Figure S6. Fluorometer parameters used to measure the fluorescence intensity of different probes.

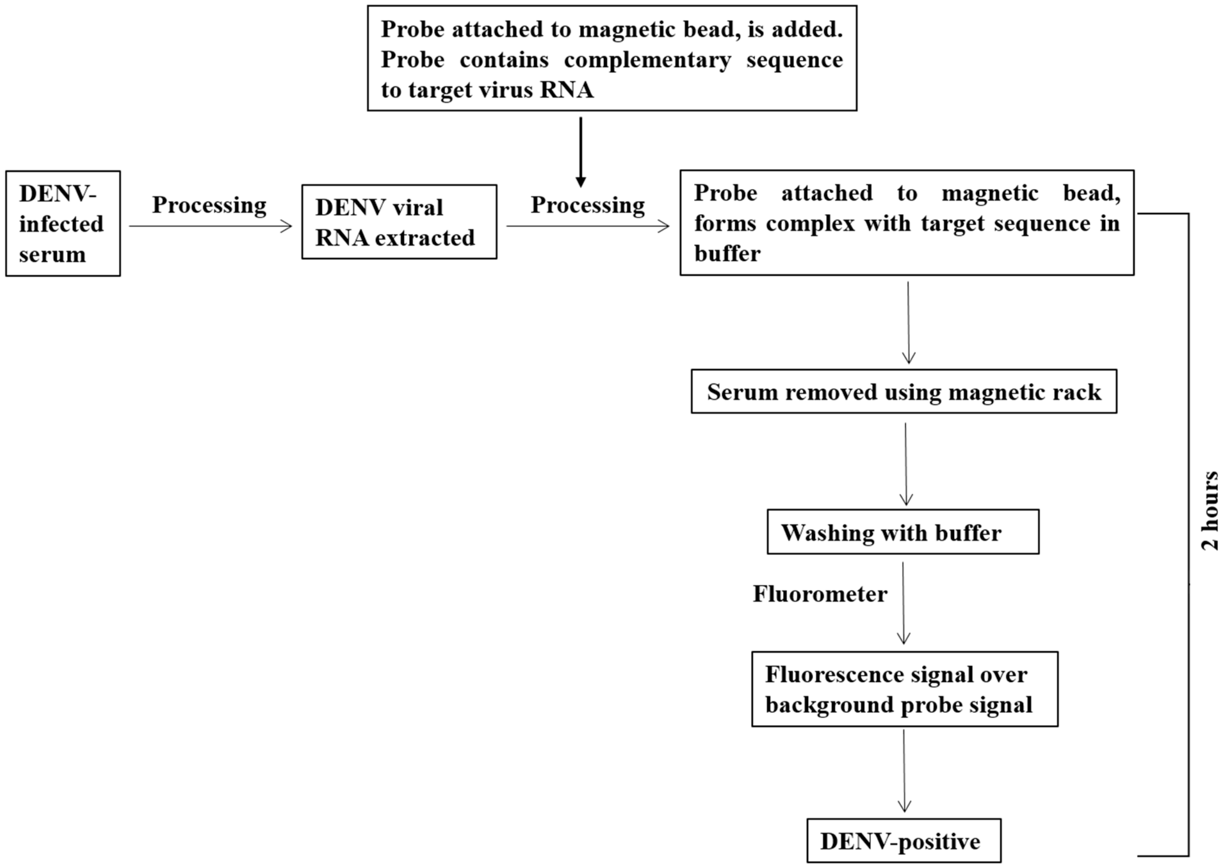

Figure S7. Schematic view of the overall process of the present invention.

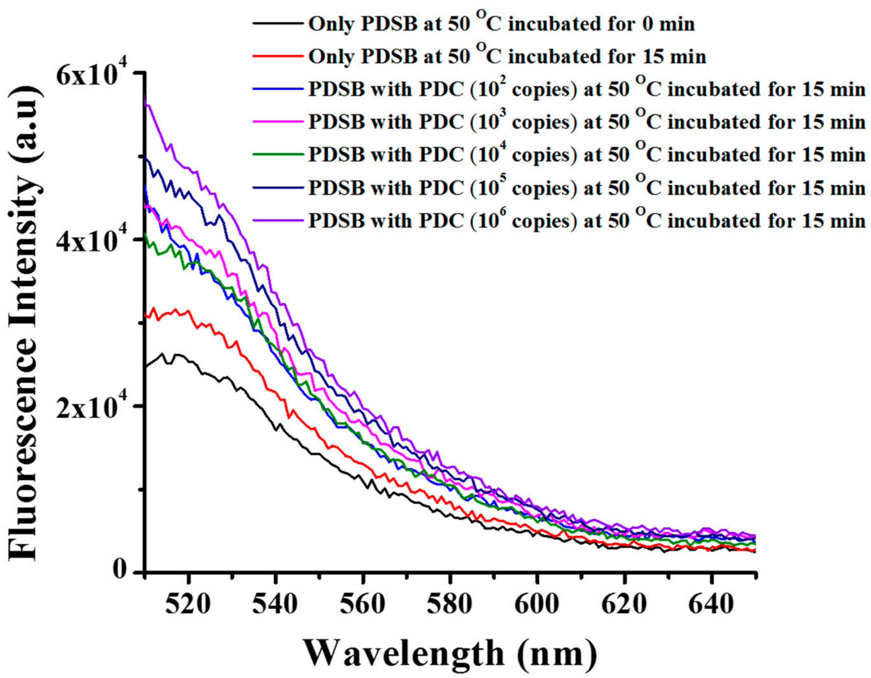

Figure S8. Fluorescence intensity measurement of PDSB probe only and PDSB probe with different concentrations of PDC target at 50⁰C for 15 min to check the sensitivity of PDSB probe.

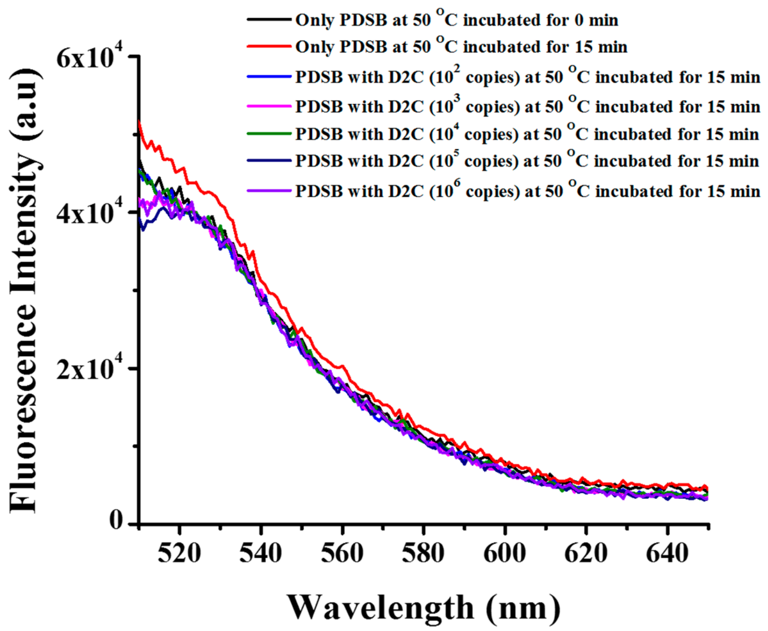

Figure S9. Fluorescence intensity measurement of PDSB probe only and with different concentrations of non-specific target (D2C) to check the specificity of PDSB probe at 50 ⁰C for 15 min.

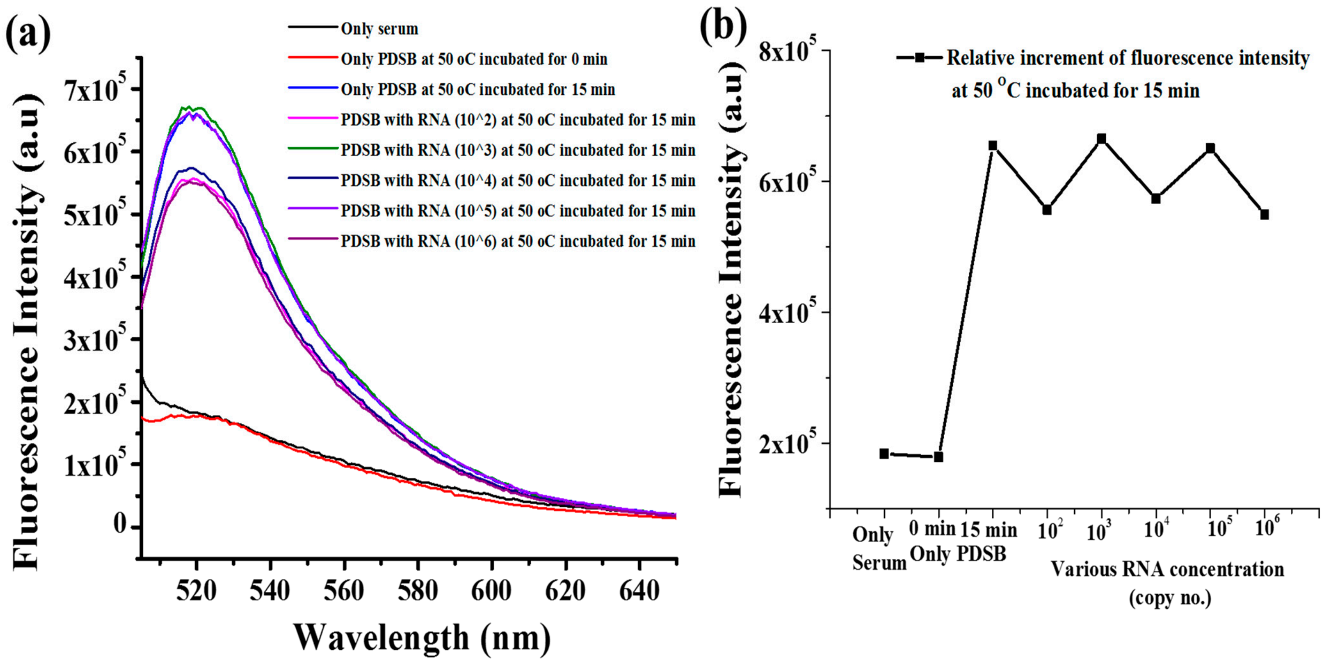

Figure S10. Fluorescence intensity measurement (a) and relative fluorescence increment (b) of PDSB probe (10^{13}) with various concentrations of DENV1 RNA at 50 °C temperature.

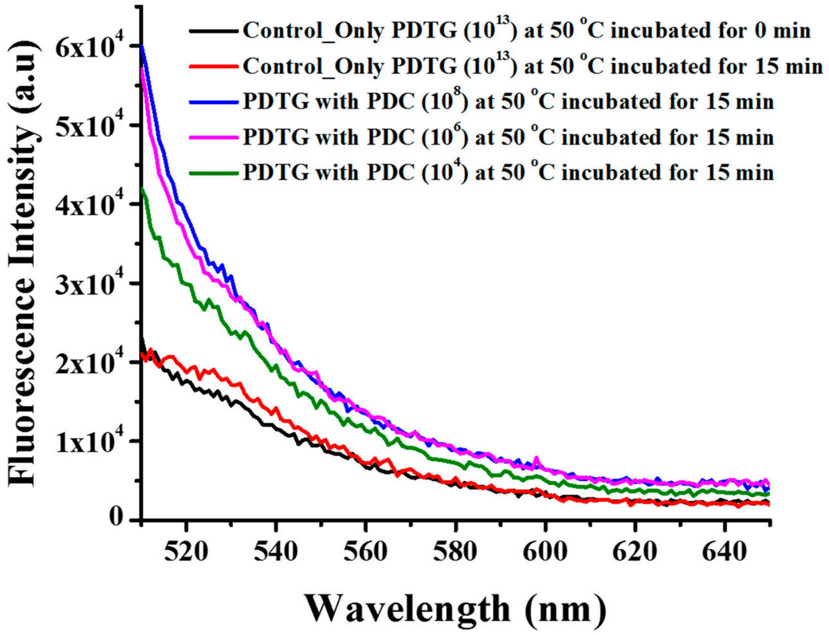

Figure S11. Fluorescence intensity measurement of only biotin tagged pan-dengue probe (PDTG) and PDTG with different copy numbers of target (PDC) at 50 °C.

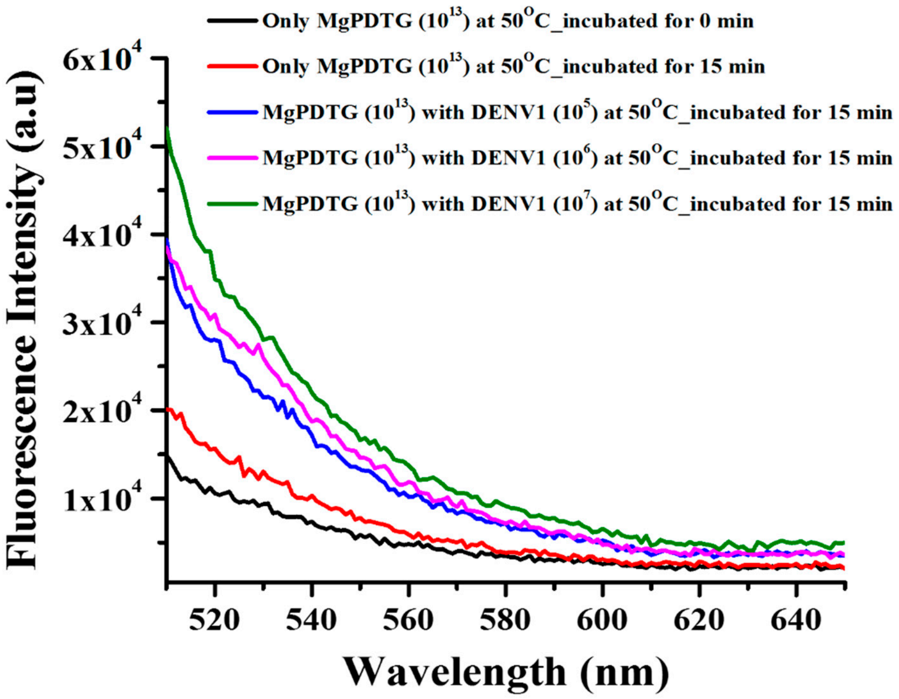

Figure S12. Fluorescence intensity measurement of biotinylated pan-dengue probe immobilized on Strep-MagBeads (Mg-PDTG) only and in presence of DENV1 target sequence at 50 °C.

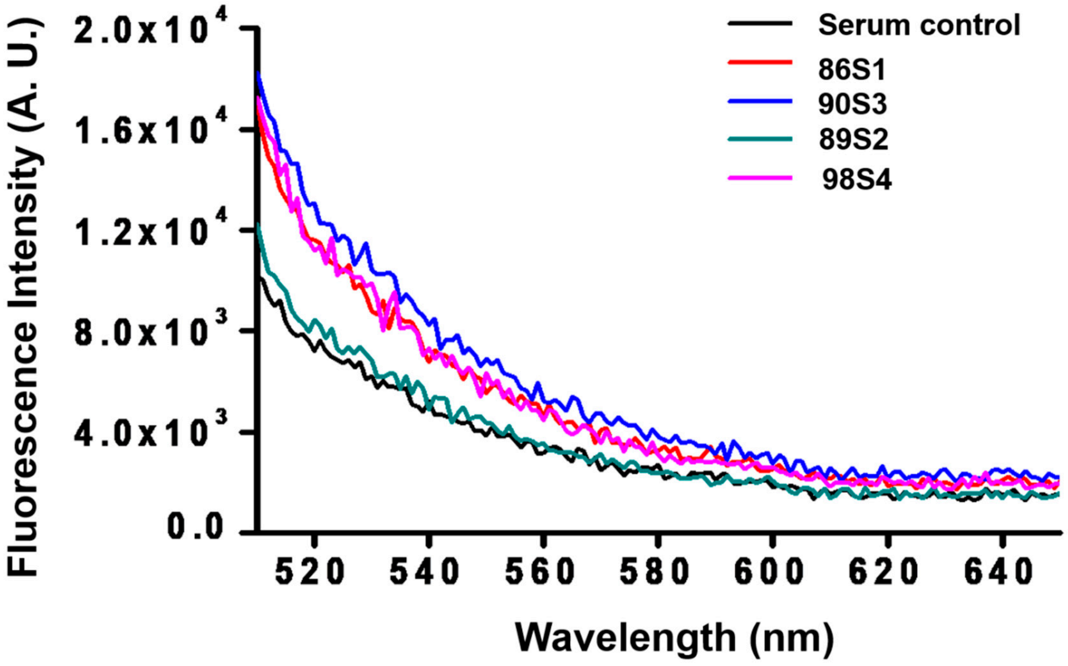

Figure S13. Detection of clinical DENV samples. Fluorescence intensity of various clinical samples measured with Mg-PDTG probe at 50 °C.

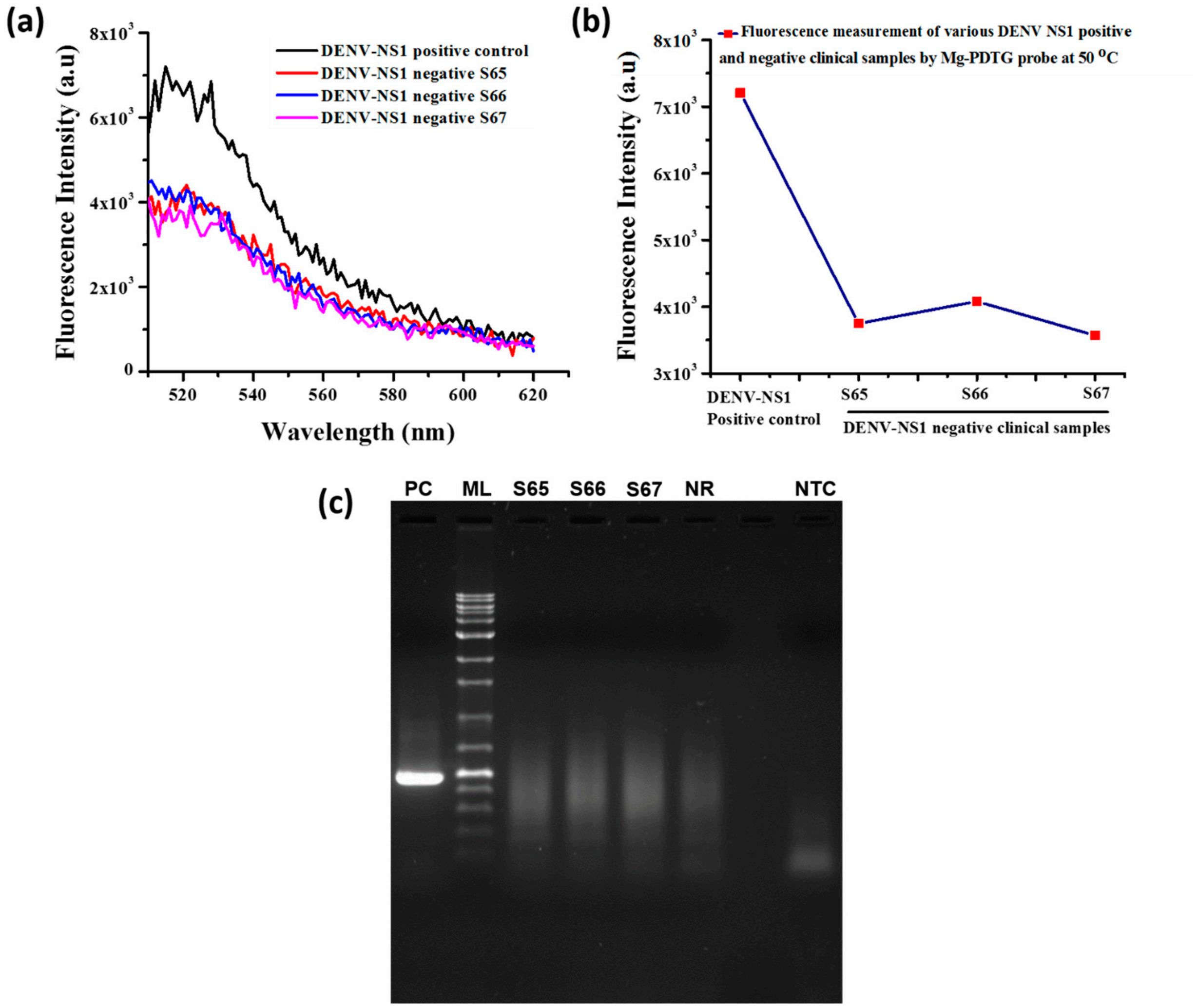

Figure S14. Detection of DENV-negative clinical samples. (a) Fluorescence intensity of a DENV NS1-positive control (PC) with three DENV NS1-negative control clinical samples (S65, S66, and S67) measured with the Mg-PDTG probe at 50 ⁰C. (b) Quantification graph showing the relative difference in fluorescence intensity between the DENV NS1-positive and DENV NS1-negative clinical samples measured with the Mg-PDTG probe at 50 ⁰C. (c) Agarose gel (1%) electrophoresis image of the DENV qRT-PCR products, obtained from RNA isolated from the DENV-NS1 positive and negative clinical samples. [PC: DENV NS1-positive control; ML: Molecular weight ladder, same as shown in Figure 3b; NTC: no-template control; NR: not relevant to this study].

	NS1-ELISA method	qRT-PCR Method	Molecular-beacon based detection
**Detection method**	Antigen	cDNA (Nucleic acid test)	RNA (Nucleic acid test)
**Serotyping**	No	Possible	Possible
**Sensitivity**	Less sensitive than the other two. Sensitivity had been reported to range from 52-86% based on different studies using different commercially available kits [1-7]	Highest sensitivity. Can detect even less than 10 copies of viral genome [8-11]	Can detect up to 10^2 copies but linearity was only perceptible from 10^4 copies and higher
**Viral load**	Not quantitative	Quantitative	Semi-quantitative
**Ease-of-use**	Easy to use	Requires trained manpower	Easy to use
**Time**	2-3 hrs	6-8 hrs	2 hrs

Table S1. Comparison between NS1-ELISA, qRT-PCR and molecular-beacon-based dengue diagnosis.

References:

(1) Solanke, V. N; Karmarkar, M. G; Mehta, P. R. Early dengue diagnosis: Role of rapid NS1 antigen, NS1 early ELISA and PCR assay. Trop J Med Res 2015,18, 95-9.
(2) Ambrose, J. H; Sekaran, S. D; Azizan, A. (2017). Dengue virus NS1 protein as a diagnostic marker: commercially available ELISA and comparison to qRT-PCR and serological diagnostic assays currently used by the State of Florida. Journal of Tropical Medicine, 2017, 8072491. doi:10.1155/2017/8072491
(3) Hermann, L. L; Thaisomboonsuk, B; Poolpanichupatam, Y; Jarman, R. G; Kalayanarooj, S et al. Evaluation of a dengue NS1 antigen detection assay sensitivity and specificity for the diagnosis of acute dengue virus infection. PLoS Negl Trop Dis. 2014, 8, e3193.
(4) Hunsperger, E. A; Yoksan, S; Buchy, P; Nguyen, V. C; Sekaran, S. D et al. Evaluation of commercially available diagnostic tests for the detection of dengue virus NS1 antigen and anti-dengue virus IgM antibody. PLoS Negl Trop Dis. 2014, 8, 3171
(5) Aryati, A; Trimarsanto, H; Yohan, B; Wardhani, P; Fahri, S et al. Performance of commercial dengue NS1 ELISA and molecular analysis of NS1 gene of dengue viruses obtained during surveillance in Indonesia. BMC Infect Dis. 2013, 13, 611.
(6) Lima, Mda. R. Q; Nogueira, R. M. R; Schatzmayr, H, G; dos Santos, F. B; Comparison of three commercially available dengue NS1 antigen capture assays for acute diagnosis of dengue in Brazil. PLoS Negl Trop Dis. 2010, 4, e738.
(7) Guzman, M. G; Jaenisch, T; Gaczkowski, R; Ty Hang, V. T; Sekaran, S. D. et al. Multi-country evaluation of the sensitivity and specificity of two commercially-available NS1 ELISA assays for dengue diagnosis. PLoS Negl Trop Dis. 2010, 4, e811.
(8) Parkash, O; Shueb, R. H. Diagnosis of Dengue Infection Using Conventional and Biosensor Based Techniques. Viruses. 2015, 7, 5410–5427.
(9) Muller DA, Depelsenaire AC, Young PR. Clinical and Laboratory Diagnosis of Dengue Virus Infection. J Infect Dis. 2017; 215, S89-S95.
(10) Lanciotti, R. S; Calisher, C. H; Gubler, D. J; Chang, G. J; Vorndam, A. V; Rapid detection and typing of dengue viruses from clinical samples by using reverse transcriptase-polymerase chain reaction. J Clin Microbiol. 1992, 30, 545–551.
(11) Waggoner, J. J; Abeynayake, J; Sahoo, M. K; Gresh, L, Tellez. Y et al. Single-reaction, multiplex, real-time RT-PCR for the detection, quantitation, and serotyping of dengue viruses. PLoS Negl Trop Dis. 2013; 7, e2116.
